# ACE-Dependent Alzheimer’s Disease: Circulating ACE Phenotypes in Heterozygous Carriers of Rare *ACE* Variants

**DOI:** 10.3390/ijms26189099

**Published:** 2025-09-18

**Authors:** Iaroslav V. Mironenko, Olga V. Kryukova, Anastasiia A. Buianova, Alexey V. Churov, Mikhail S. Arbatsky, Alyona A. Kubrikova, Yunna S. Petrusenko, Zhanna A. Repinskaia, Anna O. Shmitko, Galit A. Ilyina, Olga A. Kost, Steven M. Dudek, Irina D. Strazhesko, Ruslan I. Isaev, Elen A. Mkhitaryan, Olga N. Tkacheva, Denis V. Rebrikov, Sergei M. Danilov

**Affiliations:** 1 Institute on Aging Research, Russian Clinical Research Center of Gerontology, Pirogov Russian National Research Medical University, Ministry of Healthcare of the Russian Federation, 129226 Moscow, Russia; jarosmironen@gmail.com (I.V.M.); achurou@yandex.ru (A.V.C.); algenubi81@mail.ru (M.S.A.); kubrikova_aa@rgnkc.ru (A.A.K.); istrazhesko@gmail.com (I.D.S.); isaev_ri@rgnkc.ru (R.I.I.); melen99@mail.ru (E.A.M.); tkacheva@rgnkc.ru (O.N.T.); 2 Chemistry Faculty, M.V. Lomonosov Moscow State University, 119991 Moscow, Russia; so.b11onde@gmail.com (O.V.K.); kost-o@mail.ru (O.A.K.); 3 Genomics Laboratory, Pirogov Russian National Research Medical University, 117513 Moscow, Russia; anastasiiabuianova97@gmail.com (A.A.B.); annashmi97@gmail.com (A.O.S.); galit.ilyina@gmail.com (G.A.I.); 4 Biotech Campus LLC, 117437 Moscow, Russia; ypetrusenko@biotc.ru; 5 Department of Medicine, Division of Pulmonary, Critical Care, University of Illinois at Chicago, Chicago, IL 60612, USA; sdudek@uic.edu; 6 Center for Precision Genome Editing and Genetic Technologies for Biomedicine, Pirogov Russian National Research Medical University, 117513 Moscow, Russia; drebrikov@gmail.com

**Keywords:** angiotensin I-converting enzyme, mutations, catalytic properties, conformational changes, blood ACE, screening, Alzheimer’s disease

## Abstract

Damaging mutations of the Angiotensin I-converting enzyme (ACE) that result in low ACE levels may increase the risk of developing late-onset Alzheimer’s disease (AD)**.** We quantified blood ACE levels in EDTA-plasma from 147 subjects with 23 different heterozygous *ACE* mutations (and 70 controls) and estimated the effect of these mutations on ACE phenotype, using a set of monoclonal antibodies (mAbs) to ACE and two ACE substrates. We identified several mutations in both ACE domains (including the most frequent *ACE* mutation, Y215C), which led to decreased ACE levels in the blood, and thus could be considered as putative risk factors for late-onset AD. The precipitation of several ACE mutants (Q259R, A725P, C734Y) by specific mAbs changed significantly, and therefore, these mAbs could be markers of these mutations. Analysis of 50 of the most frequent *ACE* mutations demonstrates that more than 1.5% of the adult population may have mutations which lead to decreased ACE levels, and thus, the role of low ACE levels in the development of AD may be underappreciated. Intriguingly, statistical and cluster analyses of longevity patients revealed trends towards higher frequency of cognitive impairment among affected individuals with damaging *ACE* mutations. Systematic analysis of blood ACE levels in patients with various *ACE* mutations identifies individuals with low blood ACE levels who may be at increased risk for late-onset AD. Patients with transport-deficient *ACE* mutations theoretically could benefit from therapeutic treatment with a combination of chemical and pharmacological chaperones and proteasome inhibitors, as was demonstrated previously on a cell model of the transport-deficient *ACE* mutation Q1069R. Moreover, clinical association analysis suggests a trend linking damaging *ACE* mutations with increased risk of cognitive impairment.

## 1. Introduction

Numerous genes have been linked to the risk of Alzheimer’s disease (AD), but its etiology remains incompletely understood. Early-onset familial AD is represented by rare cases which are associated with mutations in amyloid precursor protein *APP* [1] and presenilins (*PS1* and *PS2*) [2]. However, late-onset AD is more common and a multifactorial disease associated with mutations in *APOE* and more than 30 other different genetic risk loci [3,4] involved in vascular dysfunction, immunity, inflammation, cholesterol metabolism, endocytosis, ubiquitination, etc.

One of the genes associated with AD encodes for the protein Angiotensin I-Converting Enzyme (i.e., ACE, CD143, EC 3.4.15.1) [3,5,6]. We hypothesized recently that carriers of heterozygous loss-of-function (LoF) mutations of *ACE* associated with low ACE levels could be at risk for developing late-onset AD [7]. Currently, more than 1200 *ACE* mutations are known, of which approximately 400 are predicted to be damaging by in silico analysis [7]. The frequency of these ACE LoF mutations in the general population is quite significant, up to 5% [7], which is comparable to the AD frequency in the elderly (>75 y.o.). These observations suggest that the significance of damaging *ACE* mutations (and low ACE levels caused by these mutations) for AD development is underappreciated.

The mechanism of the association between *ACE* mutations and AD could be rather direct, as ACE was shown to degrade the amyloid peptide, the major component of plaques, both in vitro [8] and in a human cell line transfected with human cDNAs encoding both the amyloid precursor protein and ACE [9]. Particularly, the N domain active center of ACE is mainly responsible for the degradation of Aβ42 [10]. Thus, experimental data suggest that lower levels of ACE correspond to higher levels of Aβ42 [9,11,12,13] and, therefore, an increased risk of AD. In addition to low surface ACE expression, there could be *ACE* mutations directly damaging the enzyme active centers, which would alter the rate of Aβ42 degradation, while some other factors could also participate [7,14].

This current study presents new ACE phenotyping data in additional carriers of *ACE* mutations using a set of monoclonal antibodies (mAbs) to ACE and two ACE substrates to estimate the effect of these mutations on different characteristics of the protein. Thus, this work is a part of the 1st stage of the planned “ACE-dependent AD” project, which is dedicated to the creation of an “Atlas of *ACE* mutations” with their phenotypic characteristics and association with AD pathogenesis. This analysis will help to identify patients with low ACE levels and, in particular, those with transport-deficient *ACE* mutations. Deep medico-genetic analysis of the families of these patients may reveal additional genetic factors in the development of AD in the carriers of a given *ACE* mutation. This 2nd stage of the project will focus on “Personalized medicine in ACE-dependent AD”. Patients with transport-deficient *ACE* mutations who have the lowest ACE levels may benefit from preventive or therapeutic treatment with a combination of chemical and pharmacological chaperones and proteasome inhibitors. This type of intervention will be the 3rd stage of the project focusing on “Therapeutic delay of AD development”. Such an approach has previously been reported using an in vitro model for another transport-deficient *ACE* mutation (Q1069R), which was causal for renal tubular dysgenesis—RTD [15].

## 2. Results and Discussion

### 2.1. Quantification of Blood ACE in Carriers of ACE Mutations

Previously, we established an approach to ACE characterization in the blood—“blood ACE phenotyping”. This approach includes measurement of ACE activity in serum or in heparinized or citrated plasma, quantification of immunoreactive ACE protein, and detection of putative conformational changes in the ACE molecule using a set of mAbs to different epitopes on the surface of the ACE globule [16,17,18,19]. However, most of the sequencing facilities operate only with EDTA-containing plasma, which makes it impossible to directly measure ACE activity, as a chelating agent causes dissociation of zinc-ions from the active centers of the enzyme. Nevertheless, ACE levels in EDTA-containing plasma can be estimated by precipitation of ACE by mAbs followed by the detection of ACE activity in ACE-mAb complexes. Using this approach, we have previously estimated blood ACE levels in 130 carriers of 27 different *ACE* mutations and 108 controls [14,20,21].

**Figure 1:**
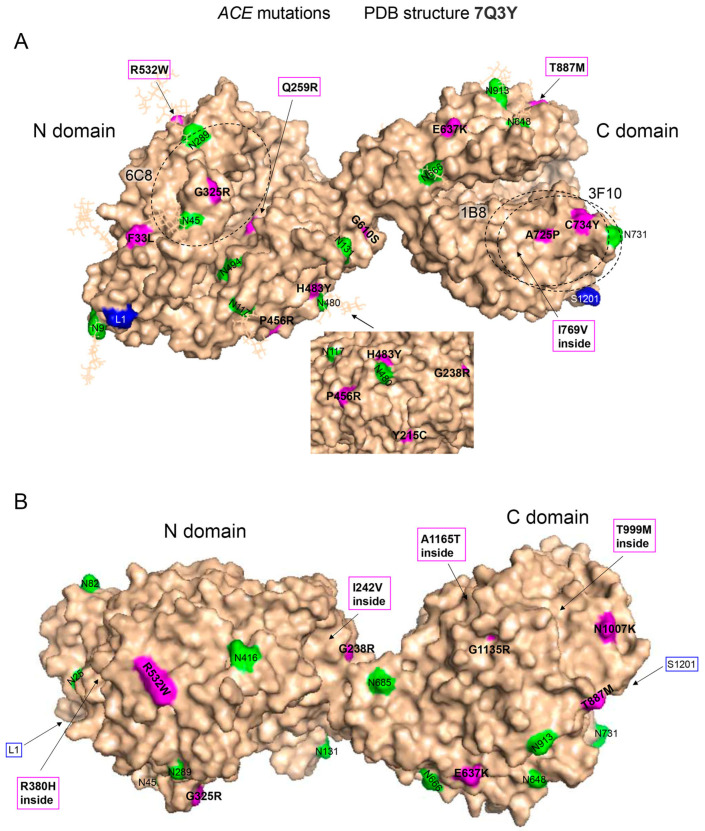
(**A**,**B**). **Molecular surface representation of mutation locations in ACE**. The position of each *ACE* mutation described in this study is shown on the Cryo-EM structure of the truncated (1–1201) human somatic ACE (PDB 7Q3Y) [23]. Key amino acids are denoted using somatic ACE numbering [22]. Specific amino acid residues colored as follows: *ACE* mutations are highlighted in magenta and additionally marked by arrows; Asn residues are putative glycosylation sites—green; highlighted in blue are the first residue in the N-domain of somatic ACE and the last visible residue in the C-terminal end of this truncated somatic ACE (marked as L1 and S1201, respectively). (**A**). Epitopes for mAbs to the N domain (mAb 6C8) and to the C domain (1B8 and 3F10)—black circles. Circle diameter is 30 Å, which corresponds to 700 Å^2^ of the area covered by each listed mAb. Insert: the mutations Y215C, G238R, P456R and H483Y—magenta; the putative glycosylation sites Asn117 and Asn480—green. (**B**) Similar localization of other relevant *ACE* mutations in the N and C domains of ACE.

In the current study, we estimated plasma ACE levels in another 147 carriers of 23 different *ACE* mutations, which were revealed during whole-exome sequencing, and in 70 controls (defined as subjects without missense *ACE* mutations). Mutations in a signal peptide (**SP1**, **SP2**, **SP3**), which is cleaved during maturation, and one mutation, **R1250W** (mature ACE numbering [22]), located in a cytoplasmic tail, could not be seen in a mature protein. The locations of the other 19 *ACE* mutations described in this study are marked with a magenta color in Figure 1A,B in a molecular model of mature two-domain somatic ACE [23]. Eleven mutations appear to be in the N domain, two of which, **R380H** and **I242V**, are hidden from the surface of the ACE globule. Of the eight mutations in the C domain, five are fully exposed on the surface, whereas the other three mutations (**I769V**, **T999M** and **A1165T**) are inside the globule and, therefore, are not visible (Figure 1A,B).

A significant achievement of this study, compared to our prior blood ACE phenotyping work [14,20,21], is that 39 patient samples from the RUSS-AGE cohort were collected both as EDTA and sera samples. Thus, these paired samples provided an opportunity to demonstrate that the data obtained by ACE precipitation with mAbs are similar for both serum and EDTA-plasma. We demonstrated that the values of ACE activity from EDTA plasma after precipitation by mAb 9B9 and determined with any substrate, ZPHL or HHL, and the values of ACE activity determined directly in serum with the same substrate were similar with a high correlation coefficient (1.02 ± 0.03) and low standard deviations of duplicates (3.5–3.9% of mean). These results confirm the accuracy of mAb-based estimations of blood ACE levels [24,25]. Thus, EDTA-containing plasma is a reliable source for the estimation of ACE levels by using mAbs in this method.

**Figure 2:**
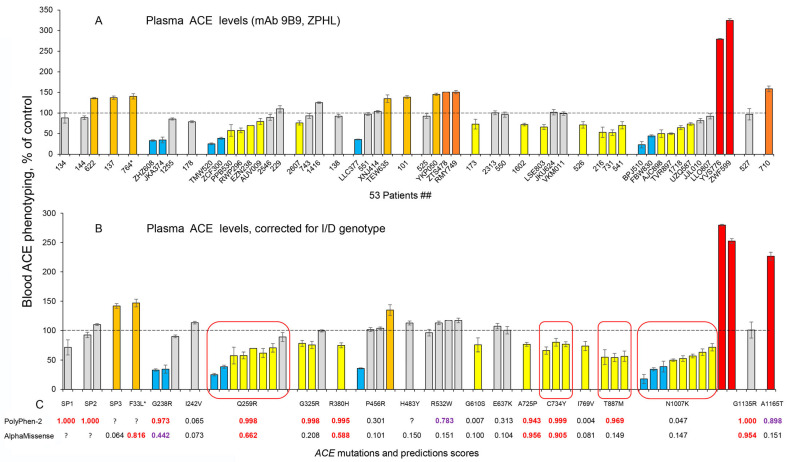
**Quantification of blood ACE levels in the carriers of *ACE* mutations**. Blood ACE protein was precipitated from EDTA-plasma by mAb 9B9 and its activity was then quantified fluorometrically (ZPHL as a substrate). (**A**) Immunoreactive ACE protein was quantified in plasma samples obtained from 53 carriers of 21 different *ACE* mutations. Asterisk indicates that ACE from subject 764 had two mutations. (**B**) Plasma ACE levels adjusted according to the donor’s genotype for the I/D polymorphism [30,31]. ACE levels for all mutations were presented as the means ± SD of several independent assessments of samples and expressed as % of ACE levels compared to the corresponding value for the pooled control plasma samples from subjects without *ACE* mutations Orange, brown and red bars indicate samples with ACE levels > 120%, 150% and 200% of controls, respectively. Yellow and blue bars—samples with ACE levels < 80% and 50% of controls, respectively. Gray bars—ACE levels between 80% and 120% of the control values. (**C**) Predictions of the potential damaging effects of 21 mutations on the ACE protein were derived from Table S1 [20] and based on Poly-Phen2 and AlphaMissense. Values shown in red are predicted to be damaging by the listed predictive engine; purple indicates the mutation is probably damaging, while values in black are predicted to be benign. * *p* < 0.01. Mutations that lead to a decrease in ACE levels are marked with red circles rounded rectangles.

ACE levels in EDTA-containing plasma samples were determined (using mAb 9B9) from carriers of 23 different *ACE* mutations, several of which were characterized by ACE phenotyping earlier [20,21], while several others are new. These ACE levels were expressed as a percentage compared to the mean blood ACE level from EDTA-containing plasma of 70 controls without *ACE* mutations (Figure 2A). It is important to note that such estimations of the effects of *ACE* mutations on blood ACE levels in a given individual are not precise enough without knowing how the ACE I/D polymorphism genotype influences both blood and tissue ACE levels [24,26,27,28]. The blood ACE levels in carriers of the DD genotype were reported to be 66% higher than in carriers of the II genotype [24,26,29]. Note, however, that the exact differences between ACE levels in these groups varied in different studies. Thus, serum ACE concentrations were reported to be 494.1, 299.3 and 392.6 (microgram/liter) for the DD, II and ID genotypes, respectively, in one study [26]; that is the ratio of DD/ID was 1.259, and that of II/ID was 0.762. In other studies, corresponding ratios of DD/ID and II/ID were reported to be 1.296–1.300 and 0.754–0.765 [30] and 1.394 and 0.680 [31]. Therefore, for our current study, ACE levels in EDTA-containing plasma samples were corrected according to a given individual’s ACE genotype, inspired by the approaches in [30,31] that suggest using correlation coefficients to properly determine relative ACE levels in sarcoidosis. We employed a similar approach to more correctly estimate the effect of *ACE* mutations on ACE levels in the blood. Specifically, we determined that the mean blood ACE level/activity for carriers of the II genotype, as determined in several studies directly or via ACE precipitation with mAb 9B9, was 72.7% of the mean in the control population, while that of subjects with the DD genotype was 128.7% [Table S1]. Therefore, blood ACE values for carriers of the II genotype were corrected using a coefficient of 0.727, and values for carriers of the DD genotype were corrected using a coefficient of 1.287. These coefficients are close in numerical values to those used in prior studies [30,31].

The results obtained with the use of these correction coefficients are presented in Figure 2B, which demonstrates the quantitative influence of each mutation on blood ACE level.

This approach provides the opportunity to identify *ACE* mutations which significantly decrease blood ACE levels. Note, however, that in the current cohort, blood samples were available from only one subject each in the case of 11 mutations, and therefore, the putative effects of these mutations (Figure 2B) on blood ACE levels should be considered as preliminary quantification. However, other blood samples collected previously were available from one subject each for 4 of these mutations, namely, **SP1**, **SP2**, G610S and **A725P** [21]. The comparison of previously collected data with current samples showed that two of these *ACE* mutations, **G610S** and **A725P**, were associated with decreased ACE levels in both cohorts of patients [21] and Figure 2B, while mutations **SP1** and **SP2** did not appear to influence blood ACE levels.

Previously, we reported that the plasma ACE levels in two out of three subjects with mutation **Q259R** were decreased [20], although one of the subjects with a low ACE level also had mutation **Y215C**, which is known to reduce ACE levels [20] and is a risk factor for AD [3]. Moreover, we found that plasma ACE in one of the patients with mutation **Q259R** was characterized by a significantly decreased ZPHL/HHL ratio (using mAb 9B9). Detailed localization of the **Q259R** mutation on the N domain of ACE clearly showed that this amino acid residue is positioned deeply inside the active center groove and located very near to catalytically important residues [20]. Thus, we suggested that the location of the **Q259R** mutation may reduce catalytic activity of the ACE N domain and be considered as a potential risk factor for AD. Here, we demonstrate that all 8 analyzed plasma samples from subjects with the **Q259R** mutation exhibited decreased ACE levels (marked by red rounded rectangles on Figure 2B). In particular, two samples had ACE levels less than 50% of the control. We speculate that the Q259R mutation might both decrease an activity of the N domain of ACE and influence on ACE traffic to the cell surface. Thus, this Q259R mutation is a strong candidate for an AD risk factor.

ACE levels were decreased in 2 out of 3 plasma samples from subjects with the **G325R** mutation (Figure 2B) in accordance with previously obtained data that 6 out of 8 plasma samples [20], and 8 out of 25 plasma samples [14], possessed decreased ACE activity. As a whole, an effect of the **G325R** mutation on ACE levels is not evident, as the carriers of this mutation demonstrated significant heterogeneity. In addition, some plasma samples from carriers of this mutation demonstrated changes in the ZPHL/HHL ratio, which indicated possible changes in ACE domain catalytic activity [14]. Therefore, the putative link between *ACE* mutation **G325R** and the mechanism of AD development requires further investigation.

Earlier, we did not find a decrease in ACE level in a single EDTA-plasma sample from a subject with the **T887M** mutation [21], but further analysis of plasma samples from 13 additional subjects demonstrated that 5 had decreased ACE levels [14]. Here in the current study, we find that another three subjects with the T887M mutation have low ACE levels (Figure 2B). Note that ZPHL/HHL ratio did not significantly change for any sample, while ACE activity was similarly decreased with both ZPHL and HHL as substrates. Thus, the **T887M** mutation can be cautiously classified as a transport-deficient mutation that may be a risk factor for AD.

Similarly, earlier, we did not find any decrease in the EDTA-plasma levels of ACE from 3 subjects with the **N1007K** mutation [21], but further analysis of another 3 subjects with this mutation demonstrated decreased plasma ACE levels [14]. New data obtained in this current study clearly demonstrate that 8 out of 10 subjects with the **N1007K** mutation have decreased ACE plasma levels (Figure 2A,B). Unexpectedly, the two other subjects with the N1007K mutation exhibited very high blood ACE levels (marked by red bars on Figure 2). One of the outliers (patient YVS776) is an 18 y.o. male with X chromosome-linked Alport syndrome 1 (OMIM 301050), which is characterized by severe glomerulonephritis. This patient is on hemodialysis, and we and others have previously demonstrated that patients with nephropathy (and glomerulonephritis) on hemodialysis have significantly elevated blood ACE levels that can be up to several fold higher [32,33,34]. Therefore, the increased blood ACE level observed in patient YVS776 with the N1007K *ACE* mutation could be completely explained by his disease. Further support for this explanation is that his healthy father (patient FBW830) has the same N1007K mutation but a dramatically decreased blood ACE level (Figure 2A,B). The other outlier with mutation N107K, subject ZWF599, may have a significantly elevated blood ACE level because this person has 3 variants of 2 additional mutations (*BMS1* and *FSIP2*)—Table S2. We found that 2388 variants of multiple genes are associated with significantly increased blood ACE levels (Buianova, 2025 in preparation), based on GWAS summary statistics of 35,559 sequenced Icelanders, whose blood ACE levels were also measured using aptamer technology [35]. However, the specific effects of the 3 mutant variants expressed by the subject ZWF599 on blood ACE levels should be validated by another independent method. Overall, subjects with the N1007K mutation generally have significantly decreased blood ACE levels (likely due to transport deficiency, similarly to the previously demonstrated Q1069R *ACE* mutation [15], and therefore, this mutation should be considered as a potential risk factor for AD. Moreover, carriers of this *ACE* mutation may benefit from the putative approach to rescue impaired trafficking of mutant ACE to the cell surface [15] to delay the development of ACE-dependent AD.

**Figure 3:**
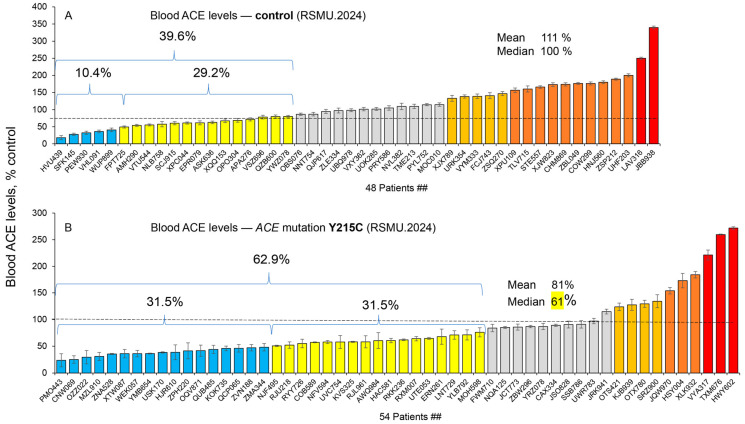
**Quantification of blood ACE levels in carriers of controls and the Y215C *ACE* mutation**. Blood ACE protein was precipitated from EDTA-plasma by mAb 9B9, and its activity was then quantified fluorometrically using ZPHL as a substrate. (**A**) The immunoreactive ACE protein was quantified in plasma samples from 48 controls. (**B**) The immunoreactive ACE protein—from 54 carriers of the Y215C *ACE* mutation. ACE levels were presented as the means +/− SD of several independent assessments of samples. The data were expressed as % of ACE levels of the corresponding value for control pooled plasma samples from donors without *ACE* mutations. Coloring is as described in the legend to Figure 2.

We have also demonstrated that a small percentage of control subjects (about 10%) have blood levels of ACE less than 50% of the mean value (Figure 3A). The percentage of patients with the most frequent *ACE* mutation associated with decreased ACE levels (defined as less than 50% of the mean value), **Y215C** (rs3730025), was much higher—about 30% (Figure 3B). As a whole, the mean ACE level in plasma from subjects with the **Y215C** mutation was significantly lower (about 80%) than the mean level in controls, and the median ACE level in plasma from subjects with this mutation (61%) was only slightly more than half of the median for controls (Figure 3B). Bearing in mind that all these subjects are heterozygotes, we suggest that the mutant allele is weakly functional. It confirms previous observations that the relatively frequent (~1%) and AD-associated **Y215C** mutation in the N domain of ACE [3,6], is truly damaging [14,20,25] and likely transport-deficient.

**Figure 4:**
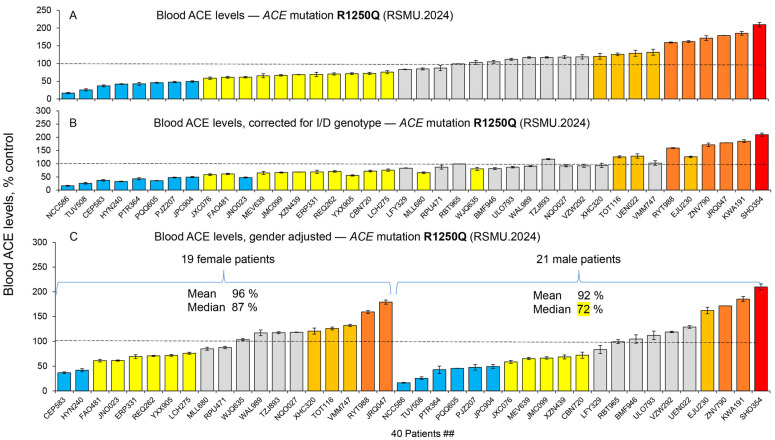
**Quantification of blood ACE levels in carriers of the R1250Q *ACE* mutation**. Blood ACE protein was precipitated from carriers of R1250Q mutation as in Figure 3. (**A**) The immunoreactive ACE protein was quantified in plasma samples from 40 carriers of the **R1250Q** *ACE* mutation. (**B**) Shown are plasma ACE levels adjusted according to the donor’s genotype for the I/D polymorphism [30,31]. (**C**) Shown are plasma ACE levels adjusted according to the donor’s gender. Data were expressed as % of ACE levels of the corresponding value for control pooled plasma samples from donors without *ACE* mutations. ACE levels for all mutations were presented as the means +/− standard deviations of several independent assessments of the individual samples. Coloring is as described in the legend to Figure 2.

We suggested previously that the reported association of mutation **R1250Q** with AD [36] might be caused by subtle conformational changes in the ACE molecule induced by this substitution due to crosstalk between the extracellular and cytoplasmic portions of ACE [7]. Earlier, we did not find a reliable effect of this mutation on ACE levels, as this value was decreased in prior studies in only 2 out of 13 plasma samples from subjects with this mutation [20] and in one out of two samples [14]. These data suggest that the mechanism of association of this *ACE* mutation with AD may be different from that for *ACE* mutation **Y215C** (which is likely due to a decrease in surface ACE expression). In the current study, we analyzed a much larger number of plasma samples from subjects with the **R1250Q** mutation (40 samples) and found that approximately 20% of them had ACE levels less than 50% of the mean value (Figure 4A,B). However, plasma ACE levels in the whole cohort of samples from subjects with this mutation only slightly differed from that of controls (mean value 94.2% ± 46.7% from control) (Figure 4A). After correction for genotype, the mean blood ACE level for this mutation showed some decrease (86.4% ± 45.4%), but it did not reach statistical significance (Figure 4B). The median value for plasma ACE levels in subjects with the R1250Q mutation was 84.1%, indicating some decrease in ACE levels; however, this decrease was much less pronounced than was observed for Y215C mutation (61.7%).

It is important to emphasize that gender appeared to play an important role in the effect of the **R1250Q** mutation on AD development: 12 of 13 heterozygous affected carriers of the minor allele of rs4980 (R1250Q-mature somatic ACE numbering) were female [36]. We have hypothesized (based on our finding that urinary ACE in men and women are differently sialylated [37]) that the sex-specific differences in tissue ACE glycosylation may be the reason for differential AD susceptibility for carriers of this mutation [7]. However, here in the current study, we observed that males exhibited a more pronounced effect of this mutation on ACE level, albeit not statistically significant, but rather a trend (Figure 4C). This observation is in apparent contrast with the report obtained earlier that females with the R1250Q *ACE* mutation are at higher risk of AD development [36]. However, we cannot exclude the possibility that the hypothesized gender effect of differential sialylation of local glycosylation sites in the N domain of ACE may be different or even opposite in terms of hydrolysis of short [37] and long substrates (as amyloid peptide Aβ42) [14].

Therefore, the association of mutations **Y215C**, **Q259R**, **G325R**, **T887M**, **N1007K**, and possibly **G325R** and **T887M** with lower levels of blood ACE could be due to a decrease in the cell surface expression of ACE caused by a transport-deficiency (similar to what we found with mutation Q1069R [15]. A change in ACE catalytic activity is another possible mechanistic explanation. Putative changes in the catalytic activity may significantly affect the rate of the hydrolysis of such substrates as Aβ42. The molecular docking of amyloid peptide Aβ42 to human ACE revealed some putatively important interactions between ACE and Aβ42, which suggest that the mutations **Q259R** and **G325R** may affect the binding and hydrolysis of Aβ42 [14]. It is worth noting, however, that the presence of a mutation and altered ACE level is not sufficient to establish a direct link between these parameters and the mechanism of AD development. There also could be mutations in the putative ACE secretase that influence ACE shedding or mutations that influence ACE expression inside the cell, etc.

Previous pharmacologic studies demonstrated that ACE inhibition may reduce beta-amyloid accumulation and neuroinflammation, supporting a protective role of ACE activity [38,39]. Two very recent studies [40,41] further support our concept of **ACE-dependent AD** and the importance of *ACE* mutations in AD development [7,14,20,21]. First, Gao et al. identified **causal** relationships of ACE gene and protein with AD indicating a protective effect of higher ACE against AD. ACE levels were strongly associated with reduced AD risk in this study (OR = 0.41) [40]. In the 2nd recent report, which was released in preprint form, Kim et al. used a cohort of 338,645 UK Bio-bank participants followed over more than 10 years to identify an association between genetically predicted low serum ACE levels and an increased incidence of AD [41].

### 2.2. Conformational Changes Induced by ACE Mutations

Valuable information about the effects of some mutations on ACE properties can be obtained by analyzing the binding of mAbs to different epitopes on the surface of the ACE globule. For this purpose, we tested the binding of mutant ACE to mAbs whose epitopes include the various mutated residues. Although individual *ACE* mutations can be identified in the sequence from any given individual, this mAb ACE binding method is valuable for studying transport-deficient *ACE* mutations. In the future, clinical trials may be conducted on carriers of these mutations to evaluate the effectiveness of a combination of chemical and pharmacological chaperones, along with proteasome inhibitors, to restore the trafficking of mutant ACEs to the cell surface. This approach was previously discussed in [7] and shown to be successful in an in vitro model of the rare transport-deficient *ACE* mutation, Q1069R [15]. An increase in the blood ACE level in patients who express mutant ACE would be an indicator of positive effects of this treatment. Thus, to assess the response to this proposed therapy, it will be essential to quantify the increased level of mutant ACE in the blood of patients.

**Figure 5:**
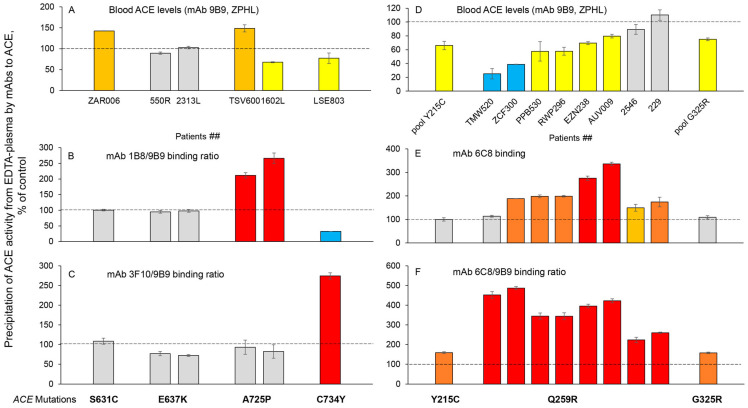
**Antibody markers for some *ACE* mutations.** Blood ACE protein was precipitated from EDTA-plasma by mAb 9B9 as well as several other mAbs, and its activity was then quantified fluorometrically with ZPHL as a substrate. ACE protein was precipitated from blood samples obtained from carriers of various *ACE* mutations using mAbs as indicated and normalized to the precipitation of ACE from the same sample using mAb 9B9 (mAbs/9B9 ratios). (**A**) Immunoreactive ACE protein was quantified in plasma samples obtained from 6 carriers of **S631C, E637K, A725P** and **C734Y** *ACE* mutations.; (**B**) Shown are the 1B8/9B9 binding ratios obtained from samples with *ACE* mutations as indicated; (**C**) Shown are the 3F10/9B9 binding ratios obtained from samples with *ACE* mutations as indicated; (**D**) ACE immunoreactive protein was quantified in plasma samples obtained from 8 carriers of the **Q259R** mutation and from two pools with mutations **Y215C** and **G325R**. (**E**) Precipitation of ACE activity in plasma samples obtained from 8 carriers of the **Q259R** mutation and from two pools with mutations **Y215C** and **G325R** by mAb 6C8 (epitope on the N domain of ACE); (**F**) Shown are the 6C8/9B9 binding ratios obtained from samples with *ACE* mutations as indicated. ACE levels for all mutations were presented as the means +/− standard deviations of several independent assessments of those individual samples. Data were expressed as % of ACE levels from the corresponding value for control pooled plasma samples from donors without *ACE* mutations. Coloring is as described in the legend to Figure 2.

We established markers for several *ACE* mutations identified in this study that are associated with a significant decrease in the ACE level and may be considered as possible risk factors for AD development. Thus, the positions of two mutations A725P and C734Y, characterized by low ACE levels (Figure 5A), are quite close to each other on the surface of the ACE C domain (Figure 1A). This area is covered by the overlapping epitopes of two specific mAbs, 1B8 and 3F10; however, these mutations caused different effects on mAb binding. While the binding of mAb 1B8 to ACE containing mutation A725P was much higher than with control ACE (Figure 5B), the binding of this same mAb to ACE containing the nearby located mutation C734Y was significantly decreased (Figure 5B). In contrast, the binding of mAb 3F10 to ACE mutant A725P was quite normal, while the binding of mAb 3F10 to ACE mutant C734Y was significantly increased (Figure 5C).

Another example of the usefulness of mAbs for distinguishing the characteristics of ACE mutants is presented in Figure 5D–F. The monoclonal antibody 6C8 can be used to identify the Q259R mutation, which is characterized by reduced ACE levels and may be a transport-deficient mutation. We also tested pooled plasma from donors with the Y215C and G325R mutations, which are characterized by lower ACE levels in the blood (Figure 5D). While the G325R mutation is located on the outer lip of the active site cleft in the N-domain of ACE, the Q259R mutation is positioned deeply inside the active center groove, very near to the catalytically important residues (Figure 1A). The position of amino acid residue 215 is almost on the opposite side of the N domain, far from the first two residues (Figure 1A, insert). The binding of mAb 6C8 was increased in most samples with the Q259R mutation (Figure 5E). However, if the ratio of mAb 6C8/9B9 binding to ACE is assessed in the plasma of donors with these mutations (Figure 5D,E), this parameter is dramatically increased in all carriers of the Q259R mutation and to a lesser extent in pooled samples of carriers of the Y215C and G325R mutations. Thus, the mAb 6C8/9B9 binding ratio can be used to estimate the concentration of ACE mutant Q259R in plasma from carriers of this mutation, such as during potential clinical trials designed to rescue impaired trafficking of this mutant ACE to the cell surface.

### 2.3. Association Analysis of ACE Mutations and Cognitive Phenotypes

To estimate the possible association of cognitive impairment and the presence of damaging *ACE* mutations, we analyzed the results of neuropsychological assessments of 200 patients of the Longevity cohort, aged 90 to 101 years (Table S3). A total of 4 out of 17 carriers and 38 out of the remaining 183 control subjects were excluded from the analysis. 19 were excluded due to significant sensory impairments that interfered with the administration of comprehensive neuropsychological testing, making the interpretation of their data unreliable, while an additional 19 control subjects were excluded because the available data were insufficient to determine the presence or absence of dementia, hippocampal dysfunction, and frontal dysfunction. Since these conditions are interdependent, participants missing information in any one of these domains were excluded from further analysis. In total, 13 *ACE* mutation carriers and 145 control subjects were included in the final analysis presented in Table 1.

Dementia was observed in 30.8% of individuals carrying damaging *ACE* mutations (4/13) and in 15.2% of non-carriers (22/145). The ARI was 15.6 percentage points (95% CI: −3.7 to 42.9). The estimated OR was 2.48, indicating that the odds of dementia were approximately 148% higher among carriers compared to non-carriers (95% CI: 0.70–8.78). The RR was 2.03, suggesting that carriers were slightly more than twice as likely to develop dementia compared to non-carriers (95% CI: 0.82–5.00). However, this difference did not reach statistical significance (*p* = 0.231, Fisher’s exact test). The association was further characterized by a Phi coefficient of +0.116 and Yule’s Q of +0.426, suggesting a weak to moderate positive relationship between *ACE* mutation status and dementia prevalence.

Hippocampal dysfunction was observed in 15.4% of individuals carrying damaging *ACE* mutations (2/13) and in 10.3% of non-carriers (15/145). The ARI was 5.0 percentage points (95% CI: −7.6 to 32.2). The estimated OR was 1.58, indicating that the odds of hippocampal dysfunction were approximately 58% higher among carriers compared to non-carriers (95% CI: 0.32–7.79). The RR was 1.49, suggesting a 49% higher probability of hippocampal dysfunction among carriers (95% CI: 0.38–5.81). This difference did not reach statistical significance (*p* = 0.634, Fisher’s exact test). The Phi coefficient of +0.045 and Yule’s Q of +0.223 indicated a very weak positive association between *ACE* mutation status and hippocampal dysfunction.

Frontal dysfunction was observed in 38.5% of individuals carrying damaging *ACE* mutations (5/13) and in 33.8% of non-carriers (49/145). The ARI was 4.7 percentage points (95% CI: −17.6 to 31.7). The estimated OR was 1.22, suggesting a 22% increase in odds of frontal dysfunction among carriers compared to non-carriers (95% CI: 0.38–3.94). The RR was 1.14, indicating a modest 14% increase in risk (95% CI: 0.55–2.35). This difference was not statistically significant (*p* = 0.765, Fisher’s exact test). The Phi coefficient of +0.027 and Yule’s Q of +0.101 indicated a negligible association between *ACE* mutation status and frontal dysfunction.

A subset of patients with frontal and hippocampal dysfunction also showed mild dementia, observed in 7.69% of individuals carrying damaging *ACE* mutations (1/13) and in 0.69% of non-carriers (1/145). These observations raise the possibility that the concurrence of dementia with hippocampal and frontal dysfunction reflects not solely the severity of Alzheimer’s disease, but rather a mixed dementia of vascular and Alzheimer’s etiology. Although the frequency was too low for robust statistical analysis, such cases warrant particular attention, and the potential association between *ACE* mutations and mixed dementia merits further investigation.

Overall, the analysis of cognitive phenotypes demonstrated consistent trends toward an association with damaging *ACE* mutations, although statistical significance was not reached.

**Figure 6:**
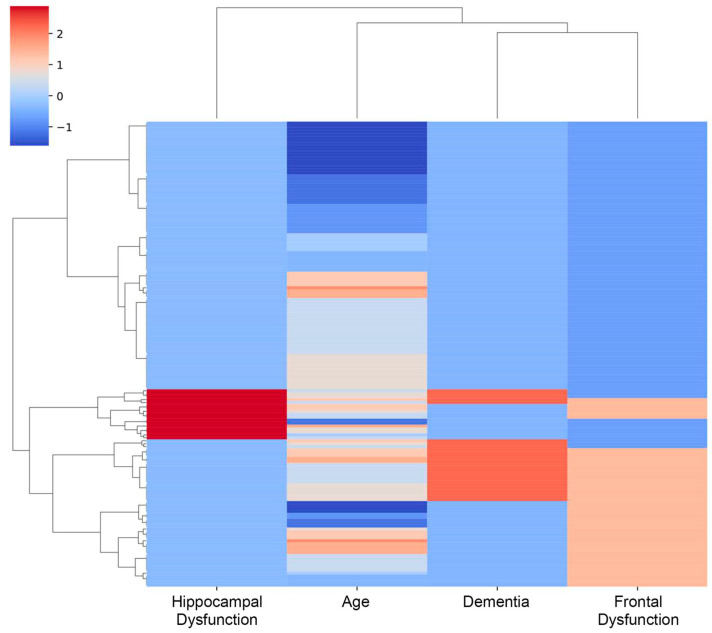
**Cluster heatmap** displaying standardized values of dementia score, frontal and hippocampal dysfunctions, and age across 158 individuals. Among the participants, 13 individuals carry *ACE* gene mutations, while the remaining 145 are non-carriers. The clustering reveals grouping based on similar neurocognitive profiles.

The clinical heterogeneity observed in both groups of the Longevity cohort (with or without *ACE* mutations) supports the use of unsupervised clustering as a more refined approach to identify patient subgroups based on multidimensional neurocognitive profiles, beyond binary mutation status. Unsupervised clustering revealed four distinct patient groups (Figure 6). These clusters did not correspond directly to *ACE* mutation status but rather captured distinct neuropsychological and clinical patterns across subgroups of patients.

Cluster 1 (*n* = 38) comprised relatively younger individuals (median age 91, IQR 90–91.8) characterized by absence of dementia, frontal and hippocampal dysfunction (all scores = 0). This cluster contained 4 individuals carrying an *ACE* gene mutation. Despite the overall enrichment of *ACE* mutation carriers in cluster 1 (10.5%), this group showed preserved cognitive function and absence of neurodegenerative signs, likely due to their relatively younger age (median 91). This suggests that the presence of the *ACE* mutation alone may not be sufficient to drive pathology in the absence of other age-related risk factors.

Cluster 2 (*n* = 53) included individuals with a median age of 95 (IQR 95–96) also with no detectable signs of dementia, frontal or hippocampal impairment (all scores = 0), indicating intact cognition. This cluster contained 3 individuals carrying an *ACE* gene mutation. Notably, Cluster 2 included cognitively intact elderly individuals and exhibited the lowest prevalence of *ACE* mutation carriers (5.7%). This relative depletion of mutations may indicate a protective role in maintaining cognitive integrity in later life.

Cluster 3 (*n* = 17) comprised the oldest patients (median age 96, IQR 95–96), marked by pronounced hippocampal dysfunction (all scores = 1). Frontal dysfunction was observed in 7 cases, and dementia was present in 5 individuals. Two *ACE* mutation carriers were assigned to this cluster. Cluster 3 exhibited the most advanced age and universal hippocampal dysfunction, along with frequent frontal impairment and dementia. It included only two *ACE* mutation carriers, but their relative frequency was the highest (11.8%).

Cluster 4 (*n* = 50) included individuals with a median age of 95 (IQR 93.25–97) with a high frequency of dementia (21/50) and frontal dysfunction (47/50), while hippocampal impairment was largely absent (all scores = 0). Four *ACE* mutation carriers were found in this cluster. Cluster 4, despite a slightly younger median age (95), was characterized by wide-spread frontal dysfunction and a high prevalence of dementia, while hippocampal function remained largely intact. Despite a mutation frequency close to that in Cluster 3 (8% vs. 11.8%), the contrasting neurocognitive profile suggests the involvement of distinct pathological mechanisms. This supports the hypothesis that *ACE* mutations may exacerbate cognitive decline in synergy with other age-related processes. Overall, this clustering helps to delineate meaningful subpopulations with distinct neurocognitive profiles, which may guide more tailored diagnostic and therapeutic approaches.

There are several limitations that may influence the significance of the results and the strength of these associations. First, the clinical diagnoses were made retrospectively, based on neuropsychological testing, without any laboratory confirmation (i.e., tTau/pTau/beta-amyloid biomarkers). Second, the modest sample size may limit the statistical power of the analysis. Third, some patients diagnosed with dementia may actually have vascular dementia, which could introduce noise into the data. In light of these findings, an expansion of the sample is planned. To ensure a more reliable diagnosis of Alzheimer’s disease in future studies, recruitment will focus on individuals with a confirmed diagnosis based on cerebrospinal fluid biomarkers.

In addition, a direct comparison of ACE levels in EDTA-plasma from control participants of the Longevity and Russ-AGE cohorts (12 individuals each, analyzed in five independent experiments) revealed that mean ACE levels in the Longevity cohort (mean age 92.6 years) were approximately 1.57-fold higher than those in the Russ-AGE cohort (mean age 41 years). We are aware that using ACE inhibitors increases ACE shedding and, thus, blood ACE levels should be elevated in patients taking ACE inhibitors [42,43]. None of the Russ-AGE controls were receiving ACE inhibitors, and only one participant in the Longevity cohort was prescribed an ACE inhibitor. Excluding this individual, the Longevity cohort (*n* = 11) still exhibited a 1.54-fold higher mean ACE level compared to the Russ-AGE controls. This observation may reflect a potential survival advantage associated with higher ACE levels in individuals reaching extreme old age [44].

## 3. Materials and Methods

### 3.1. Study Participants

Blood ACE phenotyping was performed on carriers of *ACE* mutations and controls from 2 sources: the Institute on Aging Research, Russian Gerontology Clinical Research Center, and the Center for Precision Genome Editing and Genetic Technologies for Biomedicine, both belonging to Pirogov Russian National Research Medical University, Moscow, Russia.

The protocols for collection of human blood samples and subsequent whole genome or whole exome sequencing were reviewed and approved by the Ethics Committees of the Russian Gerontology Clinical Research Center (protocol No. 59, 13 September 2022) and Ethics Committee of the Kulakov National Medical Research Center for Obstetrics, Gynecology and Perinatology (protocol No. 9, 22 October 2020). All corresponding procedures were carried out in accordance with institutional guidelines and the Code of Ethics of the World Medical Association (Declaration of Helsinki). All subjects gave written informed consent for sample collection, subsequent analysis and the use of any data for scientific purposes.

Patients’ peripheral blood and plasma (or serum) samples collected in the Russian Gerontology Clinical Research Center were obtained from two study cohorts. Samples of healthy individuals from the first cohort (*n* = 290, aged 20 to 78 years, mean age 39.9 years) were collected as a part of a larger Project RUSS-AGE (protocol No. BA06/2022, 4 July 2022) designed to study biomarkers, associated with fundamental mechanisms of aging (i.e., hallmarks of aging). For the RUSS-AGE cohort, the inclusion criteria consisted of both male and female Russian volunteers aged over 18 y.o. The exclusion criteria included acute illnesses, exacerbations of chronic diseases, infectious diseases (hepatitis C and B, including HBsAg carriage, HIV infection), significant cognitive or sensory impairments, or severe forms of non-communicable diseases (life-threatening arrhythmias, unstable angina, chronic heart failure according to NYHA, class III–IV, chronic kidney disease stages 4–5 (GFR < 30 mL/min), type 1 diabetes mellitus; type 2 diabetes mellitus with end-stage complications, systemic connective tissue diseases, stage IV osteoarthritis, history of stroke, myocardial infarction, BMI (body mass index) ≥ 40 kg/m^2^, systemic antibiotic therapy within 3 months prior to study enrollment, any invasive procedures on colon within 3 weeks prior to study enrollment; pregnancy, or lactation.

The second cohort, referred to as the Longevity cohort, comprised 200 Russian long-lived individuals (aged 90–101 years; mean age 94.3 years) recruited at the Gerontology Center to investigate genetic determinants of exceptional longevity. Inclusion criteria were male and female participants aged 90 years or older, whereas exclusion criteria were limited to inability to provide informed consent or participate in study procedures. All participants underwent a comprehensive geriatric assessment. Clinical diagnoses included cardiovascular and oncological diseases, type 2 diabetes mellitus, chronic obstructive pulmonary disease (COPD), and dementia. Retrospective evaluation of dementia and cognitive impairment was conducted by neurologists at the Russian Clinical Research Center of Gerontology. Cognitive status was assessed using a standardized battery of neuropsychological tests, including the Mini-Mental State Examination (MMSE), 5-Word Memory Test, Clock Drawing Test, Frontal Assessment Battery (FAB), and verbal fluency tests. Functional dependence was determined using the Barthel and Lawton scales. Diagnoses were established according to ICD-10 and DSM-5 criteria. An MMSE score below 23 was interpreted as dementia, while an FAB score below 14 indicated frontal dysfunction. Hippocampal dysfunction was assessed with the Dubois test, based on the difference between delayed and immediate recall. In cases with inconclusive results or sensory impairments, subgroup classification was determined by integrated analysis of all available neuropsychological measures.

Whole blood samples were collected into several vacuum tubes, containing K2-EDTA or tubes with clot activator and gel (for serum samples). One portion of the K2-EDTA samples was gently inverted 8–10 times, cooled to +4–8 °C and then stored at –80 °C. The other volume of EDTA-blood was used for separation of plasma. Plasma samples were collected by centrifugation of EDTA-blood at room temperature for 10 min at 3000 g. All serum or plasma samples of each donor were aliquoted into cryovials (LVL SAFE^®^ XLX 2000 (LVL Technologies GMBH, Creisheim, Germany) in a volume of 0.7–1 mL and stored at –80 °C. All samples (blood, serum and plasma) were collected and stored in a biobank in accordance with International Society for Biological and Environmental Repositories (ISBER) standards. For ACE phenotyping, one cryovial containing at least 0.3 mL of serum, or at least 0.4 mL of EDTA-plasma, was provided from any participant.

### 3.2. Whole Genome and Whole Exome Sequencing

Frozen blood samples were used for genomic DNA extraction and subsequent analysis on the MGI sequencing platform (MGI Tech Co., Ltd., Shenzhen, China). Whole Genome Sequencing (WGS) was performed using the DNBSEQ-T7RS platform (MGI, Shenzhen, China). Genomic DNA was isolated from the blood-containing plates using a MGIEasy Magnetic Beads Genomic DNA Extraction Kit (MGI, Shenzhen, China) according to the manufacturer’s protocol. The extracted DNA was quantified using a Qubit dsDNA Quantification Assay Kit (Thermo Fisher Scientific, Waltham, MA, USA). Each gDNA sample (1000 ng) was used to construct a genomic DNA library using the MGIEasy Fast PCR-FREE FS DNA Library Prep Set V2.0 (MGI, Shenhzen, China) according to the manufacturer’s instructions. DNA was fragmented by enzymatic fragmentation with size select step using magnetic beads. DNA end-repair and adapter ligation were conducted using the MGIEasy UDB PF Adapters-96 Kit (MGI, Shenzhen, China). The products were run on the 4200 TapeStation using the Agilent D1000 ScreenTape (Agilent, Santa Clara, CA, USA) to assess the size distribution of the libraries. They were also quantified using a Qubit dsDNA Quantification Assay Kit. The DNA fragments were circularized, and 75 fmol of ssCirDNA were amplified using rolling-circle amplification to generate DNA nanoball-based libraries, which were loaded onto a DNBSEQ-T7RS Sequencing flow cell with a DNBSEQ-T7RS High-throughput Sequencing Kit (MGI, Shenzhen, China). The library was run on a DNBSEQ-T7RS platform at paired-end 150 bp reads, with 30× coverage. Fastq files were generated using basecallLite software (ver. 1.0.7.84, MGI Tech, Shenzhen, China).

### 3.3. Bioinformatic Analysis

QC of the obtained paired fastq files was performed using FastQC [45] v0.11.9. Fastq files were trimmed using BBDuk by BBMap [46] v38.96, and trimming data were aligned to the GRCh38 genome using bwa-mem2 (v2.2.1) [47]. SAM files were converted into bam files and sorted using SAMtools [48] v1.10 to check the percentage of the aligned reads. The duplicates were marked and removed using Picard MarkDuplicates [Picard Toolkit. Broad Institute] (v2.22.4) [49].

VCF files from each of the 490 participants from Gerontology Center were obtained using VarAFT [50] v2.17 with the DbSNPv150 database. Additionally, VCF files were annotated with rsIDs from the DbSNP v156 database using bcftools [51] (v1.18). Custom scripts were used to identify variants in the *ACE* gene. The I/D polymorphism was identified based on the presence of rs4343, which is linked to an Alu repeat in intron 16. Pathogenicity of the variants was assessed using the PolyPhen-2 [52] and AlphaMissense [53].

The isolation of genomic DNA, the evaluation of its quality, preparation of DNA library, pre-capture sample pooling for enrichment following the “RSMU exome” protocol”, and sequencing of samples from 2nd cohort (RSMU samples) were carried out as described in recent studies [20,54].

### 3.4. Localization of ACE Mutations on ACE Globule

To convey the image of the ACE molecule and demonstrate the distribution of the studied mutations on (and within) the protein globule, we used the 3D structure of recombinant ACE obtained by electron microscopy (PDB number 7Q3Y [23]) and the PYMOL software package.

### 3.5. Chemicals

The ACE substrates benzyloxycarbonyl-L-phenylalanyl-L-histidyl-L-leucine (ZPHL) and hippuryl-L-histidyl-L-leucine (HHL) were purchased from Bachem Bioscience Inc. (King of Prussia, PA, USA) and Sigma (St. Louis, MO, USA). Other reagents (unless otherwise indicated) were obtained from Sigma (St. Louis, MO, USA).

### 3.6. Antibodies

The monoclonal antibodies (mAbs) to human ACE used in this study, which recognize native conformations of the N and C domains of human somatic ACE, were described previously [16,17,18]

### 3.7. ACE Activity Assay

ACE activity was measured using a fluorometric assay with two ACE substrates, 2 mM ZPHL or 5 mM HHL [24,25]. The parameter ZPHL/HHL ratio was calculated as the ratio of the rates of the hydrolysis of ZPHL and HHL by the definite ACE sample [55]. Each experiment was carried out at least 3 times, and SD values were calculated.

### 3.8. Immunological Characterization of Blood ACE

The amount of immunoreactive ACE protein in EDTA-plasma was quantified by immunoassay, in which ACE from plasma samples was captured by anti-ACE mAbs recognizing conformational epitopes on the surface of ACE molecules as described earlier [20]. For this purpose, microtiter (96-well) plates (Corning, Corning, NY, USA) were coated with anti-ACE mAbs via goat anti-mouse IgG (Invitrogen, Rockford, IL or IMTEK, Moscow, Russia) bridge and incubated with plasma samples diluted 10 times. After washing away the unbound ACE (along with EDTA and possible ACE inhibitors), precipitated ACE activity was quantified directly in the wells with ZPHL or HHL as substrates [20,24,25]. Each experiment was carried out at least 3 times, and SD values were calculated. Conformational fingerprinting of blood ACE was performed as described earlier using a set of mAbs to different epitopes of ACE [16,17,18].

### 3.9. Direct Comparison of the Levels of ACE in the Serum and Plasma of the Same Individual

We obtained the samples of both sera and plasma from 39 participants from the Russ-AGE cohort. ACE activity determined in the serum of a given patient with ZPHL or HHL as a substrate, and then it was compared with ACE activity precipitated from plasma from the same patient with mAb 9B9 and determined with the same substrate.

### 3.10. Statistical Analysis

Values of ACE activity with different substrates for each individual, as well as other parameters characterizing ACE phenotype, were expressed as means ± SD from at least 3 independent experiments with duplicates. Significance was analyzed using the Mann–Whitney test. Predictions and scores which account for evolutionary conservation and structural features were performed using four predictive tools as described for Table S1 in [7]. To identify natural subgroups of patients with similar symptom profiles and to examine the distribution of *ACE* mutation carriers within these groups, hierarchical clustering was applied. This method groups individuals not based on predefined categories but on the degree of similarity in their features. All numerical variables were first standardized (i.e., transformed to a common scale) to ensure that continuous measures such as age were comparable to binary variables, since clustering is sensitive to variable scaling. A cluster heatmap was then constructed in which patients were automatically grouped based on similarity in their neurocognitive profiles. The Ward linkage method was used, which minimizes within-cluster variance, and the coolwarm color palette was applied to visually represent the degree of expression for each variable across individuals. An analysis of clinical associations between *ACE* mutations and cognitive health in study participants was also conducted using data from the Longevity cohort (*n* = 200: 17 carriers and 183 controls), as relevant clinical information was available for this group. Absolute risk increase (ARI), odds ratio (OR), relative risk (RR), Fisher’s exact test, as well as Phi and Yule’s Q coefficients were calculated to evaluate the associations.

## 4. Conclusions

1. Precipitation of ACE by mAb 9B9 from EDTA-plasma samples from subjects with various *ACE* mutations, combined with subsequent measurement of precipitated ACE activity, provides a quantitative estimate of blood ACE levels for the purpose of identifying those associated with reduced ACE levels. We identified several mutations (including the most frequent *ACE* mutation, Y215C), which are associated with decreased ACE levels in the blood, and thus could be considered as putative risk factors for late-onset AD.

2. Partial ACE phenotyping in EDTA-plasma samples is possible through the use of mAbs to different epitopes on the ACE molecule and has established several markers for specific *ACE* mutations (e.g., mAbs 6C8/9B9 binding ratio as a marker for the transport-deficient *ACE* mutations Y215C and Q259R). This approach may be useful for future clinical trials testing therapeutic interventions to delay AD development.

3. The blood ACE phenotyping results from carriers of 23 *ACE* mutations obtained in the current study were incorporated into an updated version of Table S4 that lists 1257 missense *ACE* mutations. Blood ACE levels were estimated or quantified in samples from carriers of 91 of these mutations. From this list, Table S5 shows 50 of the most frequent *ACE* mutations. Blood ACE levels were estimated or measured for several of these and demonstrate that a substantial number are damaging mutations associated with low ACE levels. These results strongly suggest that the potential role of low ACE in the risk for development of Alzheimer’s disease is significantly underappreciated.

4. Carriers of damaging *ACE* mutations exhibited a trend toward higher rates of dementia, hippocampal dysfunction, and frontal dysfunction compared to non-carriers, indicating a possible increased risk of these cognitive impairments.

## Figures and Tables

**Table 1:** Clinical associations between carriage of *ACE* mutations and cognitive impairment.

Cognitive Impairment	*ACE* Mutation Carriers (*n* = 13)	Non-Carriers (*n* = 145)	OR (95% CI)	RR (95% CI)	ARI (95% CI)	p (Fisher’s Exact Test)	Phi	Yule’s Q
Dementia	4 (30.8%)	22 (15.2%)	2.48 (0.70–8.78)	2.03 (0.82–5.00)	15.6 pp (−3.7 to 42.9)	0.231	+0.116	+0.426
Hippocampal dysfunction	2 (15.4%)	15 (10.3%)	1.58 (0.32–7.79)	1.49 (0.38–5.81)	5.0 pp (−7.6 to 32.2)	0.634	+0.045	+0.223
Frontal dysfunction	5 (38.5%)	49 (33.8%)	1.22 (0.38–3.94)	1.14 (0.55–2.35)	4.7 pp (−17.6 to 31.7)	0.765	+0.027	+0.101

Note: OR—odds ratio; RR—relative risk; ARI—absolute risk increase.

## Data Availability

The data that support the findings of this study are available from the corresponding author upon reasonable request.

## Supplementary Materials

The following supporting information can be downloaded at: https://www.mdpi.com/article/10.3390/ijms26189099/s1.
